# Histological study on the skin structure in two mudskippers, *Periophthalmus waltoni* and *Boleophthalmus dussumieri* in relation to their terrestrial life

**DOI:** 10.1186/s42649-022-00081-2

**Published:** 2022-12-16

**Authors:** Mehran Dorostghoal, Ashraf Jazayeri, Sara Ashiri

**Affiliations:** Department of Biology, Faculty of Science, Shahid Chamran University of Ahvaz, P.O.Box: 65355-141, Ahvaz, Iran

**Keywords:** Mudskippers, *Periophthalmus waltoni*, *Boleophthalmus dussumieri*, Skin, Histology, Histometry

## Abstract

Microscopic structure of skin in two amphibious mudskipper fish; *Boleophthalmus dussumieri* Valenciennes, 1837 (*B. dussumeri*) and *Periophthalmus waltoni* Koumans, 1941 *(P. waltoni)* were investigated in relation to their lifestyle. The general structure of skin is the same among the two species. Epidermis in *B. dussumeri* was thicker significantly than *P. waltoni*. The dermal bulges were only well developed in the skin of *B. dussumeri*. Mucous cells were absent in the epidermis of *P. waltoni* but present in *B. dussumeri*. Both *B. dussumeri* and *P. waltoni* have well-developed swollen middle cells as a shared epidermal feature. The thickness of the middle cell layer of the epidermis in *B. dussumeri* was significantly greater than in *P. waltoni*. Capillaries in the dorsal and ventral parts of the body are more closely distributed to the epidermal surface in *P. waltoni* than in *B. dussumeri*. The diffusion distance in the dorsal epidermis of *P. waltoni* was less than that in the ventral epidermis of *B. dussumeri*. A comparative examination of the skin of mudskipper species suggests that, due to the more terrestrial lifestyle adopted by *P. waltoni*, the skin contributes more to respiration.

## Introduction

Oxudercinae gobies commonly known as mudskippers for their ability to move with speed and agility over the muddy substrate are amphibious teleost fishes that have fascinated scientists and naturalist. Mudskippers are very sensitive to ambient environment and this potential would be beneficial for detection of pollution levels in coastal water ecosystems (Ansari et al 2014; Santoso et al 2020). They include ten genera: *Apocryptes*, *Apocryptodon*, *Boleophthalmus*, *Oxuderces*, *Parapocryptes*, *Periophtalmodon*, *Periophthalmus*, *Pseudapocryptes*, *Scartelaos*, and *Zappa* comprising 43 species (Lauriano et al 2018). Of these, only members of four genera namely *Boleophthalmus*, *Periophthalmodon*, *Periophthalmus* and *Scartelaos* have several specializations for amphibious life, including aerial vision, aerial respiration, and terrestrial locomotion and spend time on land as part of their daily life cycle (MacNae 1968; Kumaraguru et al 2020; Mahadevan et al 2021). The species of these genera can easily move about on muddy or moist surfaces and excavate burrows in the mud and some of them are even able to climb rocks, mangrove roots or stems and all share several anatomical, physiological and sensorial specializations (Ghanbarifardi et al 2020; Kumaraguru et al 2020). It is commonly known that they spend the most of their life on land and are well-known for their air-breathing habit, when they go above the tide line. *Boleophthalmus* often stay in more aquatic environments than do *Periophthalmus* species (Zhang et al 2000). *Periophthalmus* species often lift the abdominal body by supporting it with the pectoral fins and caudal peduncle, but *Boleophthalmus* usually remain in contact with water or soft mud with the whole ventral body immersed in water (Zhang et al 2000).

Thus, many studies have been carried out on their terrestrial adaptation (Suzuki 1992). In this regard, mudskippers have evolved their skin structures to facilitate cutaneous respiration, which is the direct exchange of oxygen between the skin and air (Beon et al 2013; Lauriano et al 2018; Kim et al 2019). The skin of amphibious fishes plays a critical role in maintaining homeostasis during air exposure (Dong et al 2021). There are capillaries close to the epidermal surface (Park et al 2000) which provides an effective surface for gas exchange (Martin 2014). Histological analysis as a sensitive tool can be readily used to study the structural adaptations in the organs and provide prognostic evidence of the environmental pollutant influences (Schwaiger et al 1997). As a result, the current study was carried out to determine and compare the morphological specializations that are suitable for terrestrial life in two amphibious gobies, *Boleophthalmus dussumieri* and *Periophthalmus waltoni*. This study helps us in understanding the unique lifestyle of these species, their conservation and their use as a tool for monitoring the ecosystems.

## Materials and methods

### Fish collection and laboratory rearing

The present study was approved by the Ethics Committee of the Department of Biology, Shahid Chamran University of Ahvaz (EE/97.24.3.90357/scu.ac.ir). The fish were collected by a fishing cast net from Doragh estuary of Persian Gulf (30^°^ 27^′^ 33^″^ N, 49^°^ 00^′^ 51^″^ E) in Bandar-e Emam Khomeyni, Khuzestan, Iran from May to October 2019. The samples were transferred alive to the Histology Laboratory of Shahid Chamran University of Ahvaz, Ahvaz, Iran. The fish were kept for several weeks in an aquarium with aerated seawater at constant room temperature (25 °C), where they were free to choose between terrestrial and aquatic habitats. The fish were fed once a day throughout the study with commercial fish pellets. *B. dussumeri* Valenciennes, 1837 and *P. waltoni* Koumans, 1941 were identified according to taxonomic keys. The mean total length and body weight of *B. dussumeri* were 18.31 ± 1.47 cm and 18.89 ± 2.98 g in males (*n* = 6) and 17.1 ± 1.98 cm and 18.25 ± 4.47 g in females (*n* = 6), respectively. In *P. waltoni*, mean total length and body weight were 11.46 ± 0.97 cm and 11.17 ± 3.56 g in males (*n* = 6) and 11.03 ± 1.21 cm and 9.95 ± 3.32 g in females (*n* = 6), respectively. The sex of the fish was determined by inspecting the form of genital papilla and also based on the morphology of the gonads.

### Tissue sampling and processing

For histological studies, the fish were anaesthetized with chloroform, and then, tissue was taken from the dorsal and ventral parts of the skin of each fish and immersed in Bouin’s solution for 24 h before processing for routine paraffin embedding. The specimens were dehydrated in alcohol, cleared in xylene, and sections with 5 μm thickness were prepared using rotary microtome (Leica RM2145, Germany) and stained with haematoxylin-eosin. Microscopic images were captured using light microscopy (Olympus BH, Japan) equipped with camera (Olympus DP71, Japan).

### Histometrical analysis

For histometrical analysis, the thickness of the epidermis, superficial, middle and germinativum cell layers of the epidermis and diffusion distance, which is defined as the distance from the skin surface to the inner surface of the epidermis capillaries, were measured by Axiovision 4.5 LE software (Zeiss, Oberkochen) on digital images.

### Statistical analysis

All statistical analyses were performed in SPSS software (Version 16.0, SPSS Inc., Chicago, IL, USA). Since the t-test requires the normality of the population, the Shapiro Wilk test was used to determine the normal distribution of data. An independent sample t-test was used to compare the histometrical parameters of the skin between two species. A *p*-value less than 0.05 was considered statistically significant.

## Results

**Fig. 1:**
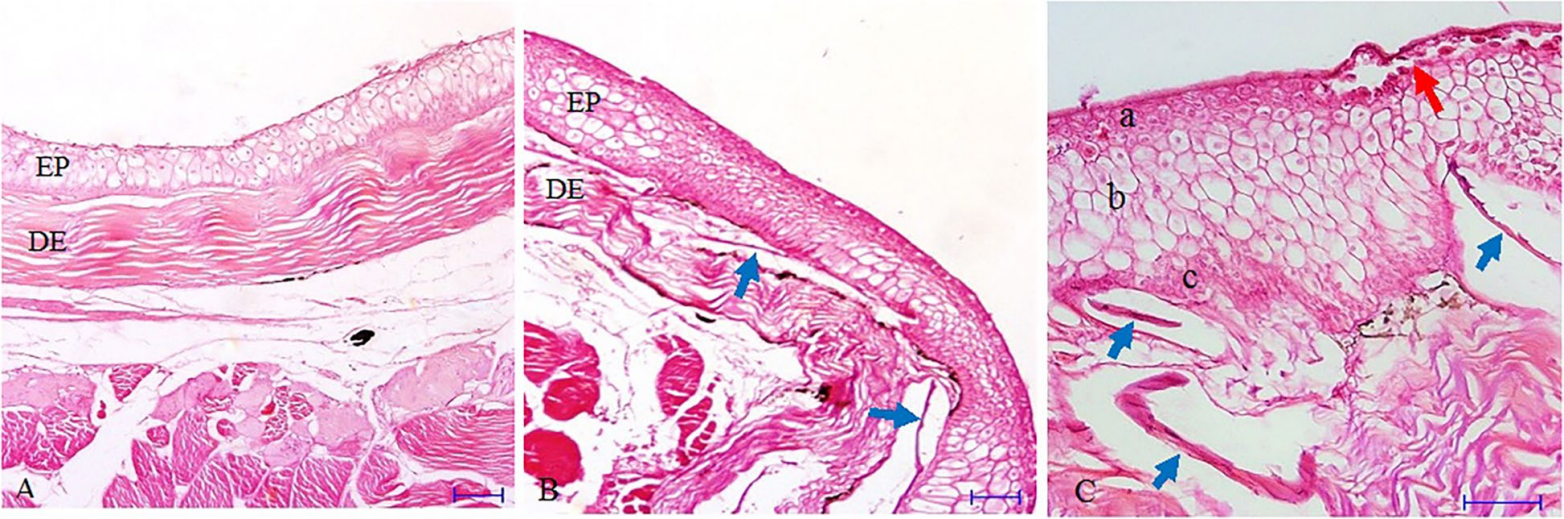
Microscopic structure of skin in *P. waltoni* (5 μm section, Hematoxylin-Eosin staining). **A** Ventral skin (Scale bar 50 μm), **B** Dorsal skin (Scale bar 50 μm), **C** Dorsal skin (Scale bar 20 μm). (EP): Epidermis, (DE): Dermis, **a** Stratum germinativum, **b** Middle layer, **c** Superficial layer, Red arrow: Blood capillary, Blue arrow: Scale

**Fig. 2:**
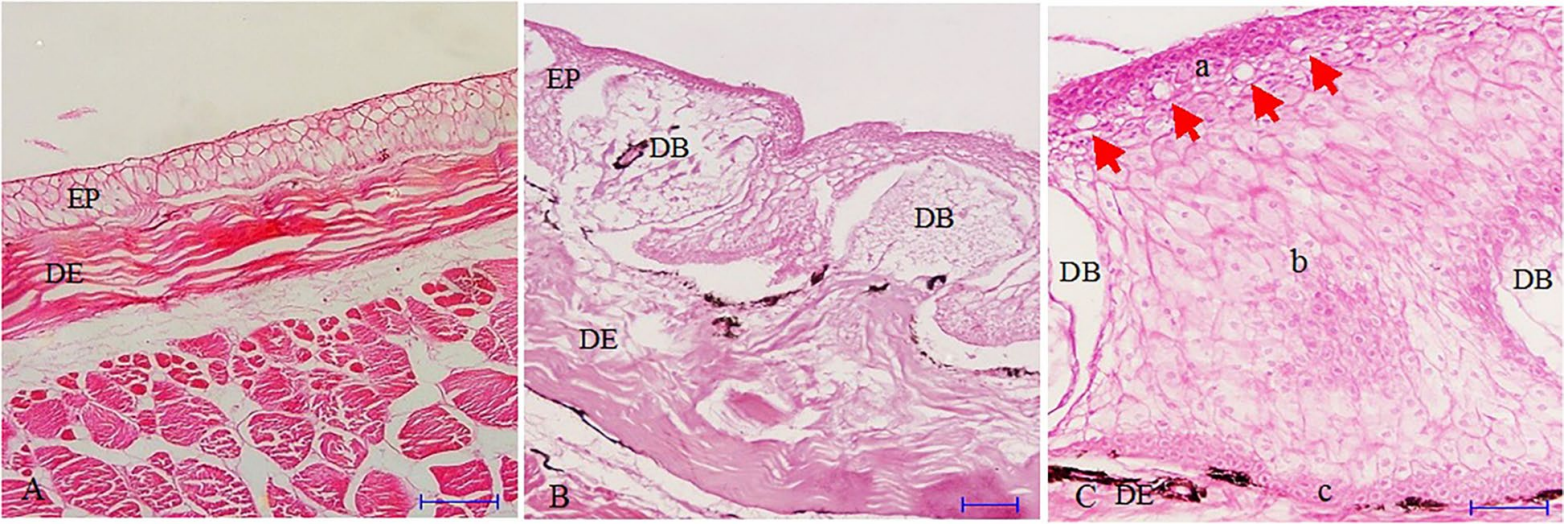
Microscopic structure of skin in *B. dussumeri* (5 μm section, Hematoxylin-Eosin staining). **A** Ventral skin (Scale bar 50 μm), **B** Dorsal skin (Scale bar 20 μm), **C** Dorsal skin (Scale bar 20 μm). (EP): Epidermis, (DE): Dermis, (DB): Dermal bulge, **a** Stratum germinativum, **b** Middle layer, **c** Superficial layer, Red arrow: Mucous cells

The skin of *B. dussumeri* and *P. waltoni* consists of the epidermis and dermis. The general structure of the skin is same between the two species. The epidermis consists of superficial, middle and basal cell layers. The superficial cell layer as the outermost layer of the epidermis consists of two to four rows of cells which vary from cuboidal to flat in shape and there are numerous blood capillaries in this layer (Figs. 1 and 2).

The middle cell layer, as the thickest stratum of the epidermis, is located between the superficial cell and basal cell layers and consists of large in size and irregular in shape cells. The middle cells are voluminous cells with a large vacuole so-called swollen cells that have a homogenous light pink cytoplasm with a clear boundary and a spherical centric blue nucleus (Figs. 1 and 2).

The basal cell layer which is arranged in one row on the basal membrane separates the epidermis from the dermis and consists of cuboidal in shape cells (Figs. 1 and 2). The dermal bulges were only well developed in the skin of *B. dussumeri* and the mucous cells were absent in the epidermis of *P. waltoni* (Fig. 2B, C).

The thickness of the epidermis was generally between 40 and 125 μm. There was a significant (*p* = 0.001) difference in thickness of the epidermis only between males (74.32 ± 12.35 μm) and females (88.97 ± 36.13 μm) of *B. dussumeri* but such a significant difference was not seen in *P. waltoni* (Table 1).

**Table 1:** The mean (±SD) thickness (μm) of the skin structural components in male and female *B. dussumeri* and *P. waltoni*

Parameter	Epidermis	Superficial layer of Ep.	Middle layer of Ep.	Germinativum layer of Ep.	Diffusion distance
			*B. dussumeri*		
Male (*n* = 6)	74.32 ± 12.35	6.98 ± 2.61	61.86 ± 14.86	5.99 ± 1.79	4.49 ± 2.63
Female (*n* = 6)	88.97 ± 36.13	6.80 ± 2.26	73.22 ± 41.83	6.55 ± 2.10	3.81 ± 1.62
*P* value	0.001	0.001	0.001	0.999	0.059
			*P. waltoni*		
Male(*n* = 6)	46.75 ± 10.18	4.36 ± 1.63	36.42 ± 10.75	5.80 ± 2.02	2.27 ± 1.33
Female(*n* = 6)	46.31 ± 14.21	6.33 ± 2.33	38.77 ± 14.77	6.27 ± 1.81	2.17 ± 1.17
*P* value	0.904	0.001	0.001	0.363	0.737

*Ep* Epidermis

The epidermis in male (74.32 ± 12.35 μm) and female (88.97 ± 36.13 μm) *B. dussumeri* were thicker significantly (*p* = 0.001) than in male (46.75 ± 10.18 μm) and female (46.31 ± 14.21 μm) *P. waltoni* (Table 2). The thickness of the dorsal and ventral epidermis were thicker significantly (*p* = 0.001) in male and female *B. dussumeri* than in male and female *P. waltoni* (Table 3).

**Table 2:** The mean (±SD) thickness (μm) of the skin structural components in male and female *B. dussumeri* and *P. waltoni*

Parameter	Epidermis	Superficial layer of Ep.	Middle layer of Ep.	Germinativum layer of Ep.	Diffusion distance
			Males(*n* = 6)		
*B. dussumeri*	74.32 ± 12.35	6.98 ± 2.61	61.86 ± 14.86	5.99 ± 1.79	4.49 ± 2.63
*P. waltoni*	46.75 ± 10.18	4.36 ± 1.63	36.42 ± 10.75	5.80 ± 2.02	2.27 ± 1.33
*P* value	0.001	0.001	0.001	0.160	0.001
			Females(*n* = 6)		
*B. dussumeri*	88.97 ± 36.13	6.80 ± 2.26	73.22 ± 41.83	6.55 ± 2.10	3.81 ± 1.62
*P. waltoni*	46.31 ± 14.21	6.33 ± 2.33	38.77 ± 14.77	6.27 ± 1.81	2.17 ± 1.17
*P* value	0.001	0.029	0.001	0.917	0.001

*Ep* Epidermis

**Table 3:** The mean (±SD) epidermis thickness and diffusion distance in *B. dussumeri* and *P. waltoni*

Parameter	Epidermis thickness (μm)					
	Males (*n* = 6)			Females (*n* = 6)		
	Dorsal	Ventral	*P* value	Dorsal	Ventral	*P* value
*B. dussumeri*	76.58 ± 9.92	72.05 ± 14.44	0.394	124.27 ± 5.92	53.68 ± 7.77	0.001
*P. waltoni*	49.95 ± 10.65	45.15 ± 9.59	0.804	57.51 ± 16.95	40.71 ± 8.18	0.001
*P* value	0.001	0.001	–	0.001	0.001	–
Parameter	Diffusion distance (μm)					
	Males(*n* = 6)			Females(*n* = 6)		
	Dorsal	Ventral	*P* value	Dorsal	Ventral	*P* value
*B. dussumeri*	2.33 ± 0.95	6.65 ± 1.89	0.001	3.53 ± 1.25	4.09 ± 1.89	0.041
*P. waltoni*	2.73 ± 1.29	2.05 ± 1.29	0.001	2.25 ± 0.94	2.13 ± 1.27	0.423
*P* value	0.811	0.001	–	0.099	0.001	–

There was a significant (*p* = 0.001) difference in the thickness of the superficial layer between males and females of both mudskippers (Table 1). The thickness of superficial layer was thicker significantly in male (6.98 ± 2.61 μm) and female (6.80 ± 2.26 μm) *B. dussumeri* than in male (4.36 ± 1.63 μm) and female (6.33 ± 2.33 μm) *P. waltoni* (Table 2).

A significant (*p* = 0.001) difference was seen in the thickness of the middle layer between males and females of both species (Table 1), and also between *B. dussumeri* and *P. waltoni* (Table 2).

The thickness of the middle layer in *B. dussumeri* (61.86 ± 14.86 μm) was significantly (*p* = 0.001) greater than in *P. waltoni* (36.42 ± 10.75 μm) (Table 2).

No significant difference was seen in the thickness of the stratum germinativum between *B. dussumeri* and *P. waltoni*, however, it was thicker in *B. dussumeri* than in *P. waltoni* (Table 2).

No significant difference was seen in the diffusion distance between males and females of both species (Table 1). The diffusion distance in the ventral skin of both male (6.65 ± 1.89 μm) and female (4.09 ± 1.89 μm) *B. dussumeri* was significantly (*p* = 0.001) higher than in male (2.05 ± 1.29 μm) and female (2.13 ± 1.27 μm) *P. waltoni* (Table 3). A significant difference was seen between the diffusion distance in the dorsal and ventral skin only in both males and females of *B. dussumeri* but not in *P. waltoni* (Table 3).

## Discussion

The skin of mudskipper fishes is adapted for terrestrial life primarily through the distribution of the mucous cells, epidermal vascularization and the presence of the dermal bulges and the middle cell layer composed of swollen cells (Zhang et al 2000). The present study showed that the thickness of the epidermis in males and females of *P. waltoni* was lower than in *B. dussumeri*. Zhang et al (2003) and Park et al (2006) reported that the thickness of the epidermis depends mainly on the thickness of the middle layer, i.e. the size and number of the swollen cells. This study showed that in *P. waltoni* and *B. dussumeri* the thinnest epidermis is in the abdominal region. The mucous cells were lacking in the epidermis of *P. waltoni* whereas found in *B. dussumeri*. Like other species of *Boleophthalmus* such as *B. boddarti* and *B. pectinirostris*, *B. dussumeri* had dermal bulges, whose function appears to be the prevention of the desiccation on exposed tidal flats (Zhang et al 2000), however such a structure was not developed in *P. waltoni*. Although the dermal bulge is not developed in *P. waltoni* as in other species of *Periophthalmus*, their entire bodies are covered by swollen middle cells. The middle cell layer likely plays an important role in defending against desiccation (Zhang et al 2000). We found the swollen middle cells in both *P. waltoni* and *B. dussumeri*. The swollen middle cells appear to be a common skin feature in mudskippers and are recognized to have a significant function not just as a barrier to water loss, but also in water storage (Yokoya and Tamura 1992; Zhang et al 2003). Several mudskipper species, including *Periophthalmus modestus* (Yokoya and Tamura 1992), *Periophthalmus magnuspinnatus* (Park et al 2003), *Periophthalmodon septemradiatus*, and *Periophthalmodon Schlosseri*, have swollen middle cells (Zhang et al 2003). On the contrary, the swollen middle cell was not found in other air-breathing fishes (Mittal and Munshi 1971; Whitear 1986; Yokoya and Tamura 1992; Graham 1997) and amphibians (Whitear 1986) and plays an important role not only as a barrier to water loss but also stored a large amount of water (Yokoya and Tamura 1992).

Our results also showed that the diffusion distance in *P. waltoni* was lower than in *B. dussumeri*. The vascularization of the epidermis is one of the similar structural features in mudskippers (Zhang et al 2003). The presence of blood capillaries in the epidermis, which is close to the outer surface, makes it possible for the gas exchange to occur between the blood cells inside capillaries and the environment. The distance of the capillaries from the skin surface is within the range of 500 μm, so the process of diffusion is quite adequate for the exchange of gases. The diffusion distance in *P. magnuspinnatus* is about 1.5 μm on average (Park et al 2000) and on the dorsal body of *B. boddarti* and *B. pectinirostris* range between 2 and 6 μm (Zhang et al 2000). This means that the skin is an efficient organ for respiration in mudskippers (Mittal and Munshi 1971). However, the diffusion distance is variable and is closely related to the life modes of mudskippers. In *B. dussumeri* which often stays in more aquatic environments, the diffusion distance is more than in *P. waltoni*. *B. dussumeri* stays in their burrows during the high tide, at which time the burrows are submerged. They emerge from their burrows when the mudflat is exposed to the air by the ebb tide, and move about on the mudflat, exposing all or a part of their bodies to the air. While in the water, they expose the head and a part of the dorsum above the water (Park et al 2003).

In *P. waltoni*, which is highly terrestrial, the capillaries in the dorsal and ventral areas of the body are more closely distributed to the surface of the epidermis rather than in *B. dussumeri*. This means that the cutaneous gas exchange in *P. waltoni* occurs more closely to the surface of the epidermis.

Moreover, the diffusion distance is different between the ventral and dorsal epidermis (Al-Kadhomiy and Hughes 1988). In *B. dussumeri* the diffusion distance in the ventral epidermis was more than in the dorsal epidermis. The ventral skin which is frequently immersed in the water or soft mud showed a greater diffusion distance. This is while in *P. waltoni* the diffusion distance in the ventral epidermis was less than in the dorsal epidermis. Tamura et al (1976) showed that the proportions of the oxygen uptake via the skin in water were 48% for *Periophthalmus Cantonensis* and 36% for *B. chinensis* while in the air the corresponding figures were 76 and 43%.

## Conclusions

According to the present study, the structural changes in the thickness of the epidermis and the middle cell layer, and in the distribution of the epidermal capillaries and the mucous cells represents the diverse lifestyles of mudskippers. Our findings show that in *P. waltoni* that having a more terrestrial lifestyle the skin makes a larger contribution to respiration. Future studies could be used not only to investigate the biological features and the complexity of the mudskippers, but also to evaluate their ecotoxicological significance for biomonitoring of coastal pollution.

## Data Availability

The data used to support the findings of this study are available from the corresponding author upon request.

## Abbreviations

*B. dussumeri*: *Boleophthalmus dussumieri*
*P. waltoni*: *Periophthalmus waltoni*
*B. boddarti*: *Boleophthalmus Boddarti*
*B. pectinirostris*: *Boleophthalmus pectinirostris*
*B. chinensis*: *Boleophthalmus Chinensis*
EP: Epidermis
DE: Dermis
